# Where Women Give Birth Is Changing in Sub‐Saharan Africa: Evidence From 30 Countries Using DHS Data

**DOI:** 10.1155/jp/2785076

**Published:** 2026-03-23

**Authors:** Alex Bawuah, Michael Sarfo, Godness Biney, Sanni Yaya

**Affiliations:** ^1^ School of Global Studies, Faculty of Social Science, University of Sussex, Brighton, UK, sussex.ac.uk; ^2^ School of Human and Health Sciences, University of Huddersfield, Huddersfield, UK, hud.ac.uk; ^3^ Department of Biostatistics and Epidemiology, School of Public Health and Health Sciences, University of Massachusetts Amherst, Amherst, Massachusetts, USA, umass.edu; ^4^ The George Institute for Global Health, Imperial College London, London, UK, imperial.ac.uk

**Keywords:** birth, home delivery, pregnancy, reproductive health, sub-Saharan Africa, women′s health

## Abstract

**Introduction:**

In sub‐Saharan African region, high‐maternal mortality is high due to complications of labor, delivery as well as low patronage of antenatal care and skilled birth attendants (SBAs), poverty and poor health‐seeking behavior. Although considerable studies exist on drivers of choice of birth locations, there is a paucity of current, nationally representative samples from various SSA countries examining factors influencing birthplace choices. This study addresses this gap by employing a recent dataset to assess the determinants of changing birth locations in SSA.

**Methods:**

This cross‐sectional study used the most recent Demographic and Health Survey (DHS) data from 30 sub‐Saharan African countries collected between 2014 and 2024, comprising 61,240 women aged 15–49. Two outcomes were examined: (i) any change in childbirth location between the two most recent births, and (ii) a shift from home to health‐facility delivery. Two binary logistic regression models were fitted to identify factors associated with each outcome, with checks for multicollinearity conducted using variance inflation factors and tolerance indices. Survey design was accounted for in our regression analyses (sampling weights and clustering).

**Results:**

Overall, 13.59% (*n* = 8320) of women changed their childbirth location between their two most recent births, and more than half of these women (54.35%) shifted from home to a healthcare facility. The highest change is observed in Uganda where 20.36% (95% CI; 19.07–21.66) changed their location of childbirth, and the lowest is observed in Burkina Faso where only 5.24% (95% CI; 2.99–7.48) changed their location of childbirth. The probability of changing birth location from home to a healthcare facility increased with the level of education, with those with higher education having a higher likelihood of changing from home to a facility (AOR = 2.76, 95% CI: 1.53–4.97) compared with those with no education. The odds of changing birth location from home to a healthcare facility increase with wealth status, particularly for women in the richest category (AOR = 2.31, 95% CI: 1.79–2.97) relative to those in the poorest category. Women in rural areas are 34% less likely to change from home to a facility compared with those in urban areas (AOR = 0.66, 95% CI: 0.57–0.76).

**Conclusion:**

Our study highlights significant disparities in changes in childbirth locations across SSA countries, driven primarily by education, wealth, and rural–urban residence. Although countries like Malawi and Zimbabwe demonstrate successful strategies for promoting facility‐based deliveries, others like Chad and Burkina Faso face persistent barriers. Addressing these disparities requires targeted interventions, including expanding rural healthcare infrastructure and implementing pro‐poor healthcare policies. Future research and program designs should prioritize longitudinal assessments of these determinants to tailor interventions for specific country contexts.

## 1. Introduction

According to the 2020 World Health Organization (WHO) report, maternal death occurs nearly every 2 min around the world [1]. The report estimates that 27,800 women died during and following pregnancy and childbirth, with approximately 95% of all cases occurring in low and middle‐income countries. Sub‐Saharan Africa (SSA) accounts for a staggering 70% of all cases [1]. The increasing incidence of maternal mortality in the world, specifically in the SSA region, is one of the core factors for the establishment of the Sustainable Development Goals (SDGs) in 2015, with Goal 3 aiming to promote reproductive health among women [2, 3]. Particularly, Goal 3.1 mandates countries to reduce the maternal mortality ratio to less than 70 deaths per 100,000 live births by 2030 [3]. Globally, from 2007 to 2017, there was a significant reduction in maternal mortality rate by 38%; quite remarkably, in SSA, there was nearly 40% in the same period [1, 4].

Notwithstanding, maternal mortality remains one of the leading causes of death among women in Africa, with women having a 1‐in‐36 lifetime risk of maternal mortality [5, 6]. In the SSA region, high‐maternal mortality occurs due to complications of labor, delivery, or during the few 24 h after birth [7]. Other factors are attributed to low patronage of antenatal care and skilled birth attendants (SBAs), poverty, poor health‐seeking behavior, direct and indirect obstetric causes such as hemorrhage, sepsis, immune system disorders, unsafe abortions, obstructed labor, diabetes, and hypertensive disorders [5, 8–10]. Although Gebreegziabher et al. [11] and Ossai et al. [12] have asserted that birth delivery in the hospital setting by skilled attendants and with available resources significantly reduces maternal mortalities, stillbirth rate, and neonatal morbidity, the proportion of place of delivery, whether at home or at the health facility, varies from countries [13]. According to the 2021 UNICEF report, globally, 84% of births occurred in health facilities [14]. Of this, nearly all newborns (99%) from developed countries were born in health facilities. Conversely, only 64% of child deliveries in SSA occurred in health facilities with skilled birth attendance.

An array of factors has been noted to influence the choice of birthplace among women. For instance, Olubodun et al. [13] in their study in Nigeria found that 41% of women delivered their last babies in the hospital. It further asserts that age, ethnicity, mass media, gender, level of education, proximity to health centers, and geographical locations were the main determinants of hospital deliveries among the study population. In a similar vein, Montagu et al. [15] found in their study an increase in health facility deliveries within the space of 15 years among participants from wealthy homes in Asians with an inverse slight decline among the rich population in Central and West Africa. The observed paradigm shift among the study population is attributed to factors such as the availability of national policies, the cash‐and‐carry system, urbanization, and social norms.

A previous study among 18 SSA countries by Straneo et al. [16] on the use of childbirth care in hospitals reveals that a minority of rural inhabitant women used hospitals for deliveries. Prominent among the findings is the disproportionate use of hospitals by women from wealthy homes relative to those from poor backgrounds, as well as by women with high parity compared with those having their first child. With Africa grappling with noncommunicable diseases, infectious diseases, and the reemergence of diseases [17], alongside recovering from the strain of the recent pandemic and being just 5 years away from achieving the Sustainable Development Goals, particularly Goal 3, it is imperative to explore both modifiable and nonmodifiable factors influencing the choice of birthplace among women of reproductive age in SSA, a region heavily burdened by inequalities. Despite existing studies on static drivers of birth locations [8, 18–20], little is known about the dynamics of location shifts for successive births across the broader SSA region. Specifically, there is a lack of diverse, nationally representative evidence examining why women switch from home to facility‐based care or vice versa between pregnancies. Therefore, to achieve this study′s objective and to address the existing gap in the literature through the use of recent multicountry Demographic and Health Surveys (DHS) data from SSA, this study is conceptually grounded in Andersen′s Behavioral Model of Health Services Use [21, 22]. This framework argues that health‐seeking behavior is not random but is influenced by three categories of determinants, which encompass predisposing characteristics (such as age and marital status), enabling resources (including wealth, education, and employment) and need factors (such as reproductive history and birth intervals). By incorporating this framework, we aim to extend beyond descriptive analysis to explore how these structural and individual factors predict the decision to change childbirth locations.

## 2. Methods

### 2.1. Data Source

This cross‐sectional study used the most recent data from the DHS of 30 countries in SSA conducted from 2014 to 2024. The countries included are Angola, Benin, Burkina Faso, Burundi, Côte d′Ivoire, Cameroon, Ethiopia, Gabon, Ghana, Gambia, Guinea, Kenya, Liberia, Lesotho, Madagascar, Mali, Mauritania, Malawi, Mozambique, Nigeria, Rwanda, Sierra Leone, Senegal, Chad, Togo, Tanzania, Uganda, South Africa, Zambia, and Zimbabwe. The DHS is a nationwide survey that is conducted in over 85 low‐ and middle‐income countries worldwide and follows a consistent protocol and terminology across all countries [23]. It employs a structured questionnaire to gather information on various indicators of health, including maternal and child health, fertility, family planning utilization, morbidity, and mortality [23]. The DHS uses a two‐stage sampling technique to collect data, starting with the selection of enumeration areas based on each country′s sampling frame, followed by the selection of households from each enumeration area. Detailed information on the sampling and data collection methods can be found in the work of Aliaga and Ren [24]. The study employed the women′s dataset (IR file) from the DHS. Retrospective birth histories were analyzed. The analysis for this study was restricted to the two most recent live births of the women. Thus, for this study, the sample was limited to women with at least two live births. Also, twins or multiple births were considered one birth. The total sample size for the study is 61,240 women.

### 2.2. Variables

In the survey, the women were asked about the place of birth for all their live births. We categorized the place of birth into two: home and healthcare facility, where childbirth at a healthcare facility included births that had taken place at a public health facility, NGO or trust hospital, or private hospital/maternity home/clinic, and home births included live births which took place at respondents′ home or other homes. Two outcomes of interest were defined. The first outcome captured any change in childbirth location between the two most recent births, regardless of direction, and was coded as 0 = “no change” (home‐to‐home or facility‐to‐facility) and 1 = “changed” (home‐to‐facility or facility‐to‐home). The second outcome captured a directional change from home to a health facility, restricted to women whose earlier birth occurred at home, and was coded as 0 = “home‐to‐home” and 1 = “home‐to‐facility.” This second outcome, therefore, measures the net shift toward facility‐based delivery.

The study included 10 explanatory variables. They include age (15–19, 20–24, 25–29, 30–34, 35–39, 40–44, and 45–49), education (no education, primary, secondary, and higher), residence (urban and rural), wealth (poorest, poorer, middle, richer, and richest), marital status (never in a union, married, living with a partner, widowed, divorced, and separated), employment status (employed and unemployed), ever terminated a pregnancy (no, yes), birth interval (< 24, 24–36, and > 36 months), frequency of watching television (not at all, less than once a week, at least once a week, and almost every day), and frequency of listening to radio (not at all, less than once a week, at least once a week, and almost every day). These variables were selected based on their availability in the DHS dataset and also on a conceptual framework grounded in Andersen′s Behavioral Model of Health Services Use and related models of maternal healthcare utilization, which posit that health‐seeking behavior is shaped by predisposing characteristics, enabling resources, and perceived or evaluated need for care [21, 22]. In this framework, age, marital status, and birth interval capture women′s reproductive life course and prior childbirth experience, which influence risk perception, confidence, and preferences for facility delivery. Education, wealth, and employment reflect socioeconomic position and enabling resources that determine women′s ability to afford, access, and navigate healthcare services. Place of residence reflects geographical access and availability of healthcare infrastructure, particularly for skilled birth attendance. Exposure to mass media (radio and television) represents access to health information and public health messaging that can influence awareness of the benefits of institutional delivery and perceived norms around childbirth. Finally, the history of pregnancy termination reflects prior interaction with the healthcare system and reproductive health‐seeking behavior, which can shape subsequent use of maternity services. These pathways are well‐established in the maternal health literature and have been widely applied in analyses of DHS data [25, 26].

### 2.3. Data Analysis

The data were analyzed with STATA Version 18. Descriptive statistics were used to describe the sample and analyze the patterns of changes in childbirth location. Two binary logistic regression models [27] were employed to achieve the study′s objective. The first model (Model 1) assessed the factors associated with changing the location of childbirth, and the second model (Model 2) assessed the factors associated with changing the location of childbirth from home to a health facility. The survey design (sampling weights and clustering) was considered in the regression analyses. Prior to the regression analysis, a diagnostic test for collinearity between the independent variables was done by looking at the variance inflation factor (VIF) and tolerance indices (TI) of the independent variables. VIFs above five and TIs less than 0.20 are considered signs of multicollinearity [28]. The result of the diagnostic test is provided in Table S1.

### 2.4. Ethical Consideration

We used a secondary dataset that is freely available to the public from the DHS Program; therefore, no ethical approval was requested. The dataset is anonymized, and more details regarding its ethical standards are available at http://goo.gl/ny8T6X.

## 3. Results

### 3.1. Descriptive Statistics of the Samples

Table 1 presents the results of the descriptive summary of the sample. It shows that a higher proportion of the women are aged between 25 and 29 (29.41%). Also, a substantial proportion of respondents had no formal education (43.72%), never in a union (75.76%), in the poorest wealth category (27.46%), live in rural areas (71.40%), do not listen to radio (52.17%), do not watch television (67.46%), currently employed (61.26%), never terminated a pregnancy (87.57%), and have birth intervals between 24 and 36 months (48.51%).

**Table 1:** Demographic characteristics and patterns of changes/shifts in childbirth location among individuals.

		Changed versus did not change			Changed from *H* to *F* versus did not change (*H* to *H*)		
Variables	Total *N* = 61240	% Changing *N* = 8320	% Not changed *N* = 52,920	*ꭓ*2*p* value	% Changing from *H* to *F* *N* = 4522	% Not changing *H* to *H* *N* = 20,016	*ꭓ*2*p* value
**Age**				< 0.001			0.164
15–19	3.74	4.46	3.63		3.41	4.25	
20–24	23.67	26.47	23.23		23.07	22.54	
25–29	29.41	28.79	29.50		29.35	28.85	
30–34	21.84	20.17	22.11		21.74	21.67	
35–39	14.27	13.34	14.41		14.48	14.27	
40–44	5.69	5.42	5.74		6.26	6.42	
45–49	1.38	1.36	1.39		1.70	2.01	

**Education level**				< 0.001			< 0.001
No education	43.72	43.16	43.81		47.46	66.70	
Primary	32.70	36.54	32.10		35.23	25.04	
Secondary	20.70	19.27	20.93		16.45	7.99	
Higher	2.87	1.03	3.16		0.86	0.26	

**Marital status**				< 0.001			< 0.001
Married	3.28	3.65	3.22		3.16	2.33	
Never in union	75.76	73.80	76.07		76.78	80.57	
Living with a partner	15.58	16.53	15.43		14.90	12.49	
Widowed	1.00	1.19	0.97		1.22	1.02	
Divorced	1.34	1.27	1.35		0.95	1.14	
Separated	3.04	3.56	2.96		2.99	2.45	

**Wealth**				< 0.001			< 0.001
Poorest	27.46	30.13	27.04		30.21	39.91	
Poorer	22.97	25.19	22.62		26.34	27.37	
Middle	20.36	22.54	20.02		21.76	18.19	
Richer	16.51	14.71	16.80		14.15	11.06	
Richest	12.70	7.43	13.52		7.54	3.48	

**Residence**				< 0.001			< 0.001
Urban	28.60	24.31	29.27		22.53	13.98	
Rural	71.40	75.69	70.73		77.47	86.02	

**Frequency of listening to radio**				0.001			< 0.001
Not at all	52.17	50.56	52.43		51.48	67.58	
Less than once a week	18.40	19.84	18.18		19.26	13.77	
At least once a week	27.36	27.42	27.36		27.33	17.08	
Almost every day	2.06	2.18	2.04		1.92	1.57	

**Frequency of watching television**				< 0.001			< 0.001
Not at all	67.46	70.26	67.02		72.16	84.64	
Less than once a week	11.58	12.24	11.47		12.23	7.07	
At least once a week	18.58	15.08	19.13		13.53	7.16	
Almost every day	2.38	2.42	2.38		2.08	1.13	

**Employment status**				0.212			< 0.001
Unemployed	38.74	38.12	38.84		37.93	42.96	
Employed	61.26	61.88	61.16		62.07	57.04	

**Ever terminated a pregnancy**				0.003			< 0.001
No	87.57	86.56	87.72		86.60	89.51	
Yes	12.43	13.44	12.28		13.40	10.49	

**Birth interval (months)**				< 0.001			< 0.001
< 24	27.81	24.75	28.29		22.09	26.84	
24–36	48.51	50.81	48.15		49.89	49.62	
> 36	23.68	24.45	23.56		28.02	23.55	

Abbreviations: F, facility; H, home.

### 3.2. Pattern of Changes/Shift in Childbirth Location Among Individuals

**Figure 1:**
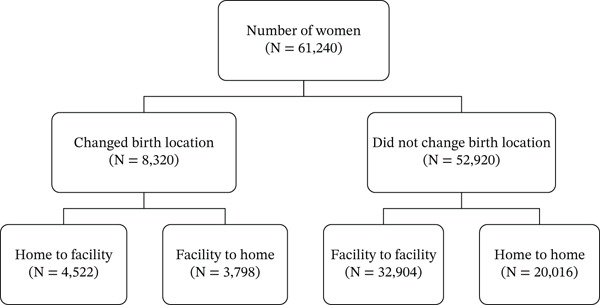
Pattern of changes in childbirth location among individuals.

Figure 1 presents the pattern of changes in childbirth location among individuals. It shows that 8320 (13.59%) of the women changed their childbirth location for their last birth. Of the women who changed childbirth location, 4522 (54.35%) moved from home to a facility, whereas 3798 (45.65%) moved from a facility to a home (their demographic and socioeconomic characteristics are presented in Table S2). For the 52,920 women who did not switch the location of childbirth, most of them (32,904 [62.18%]) were from facility to facility. The results from Table 1 revealed that most of the women who changed the location of childbirth as well as those who switched from home to a healthcare facility are aged between 25 and 29, have no education, have never been in a union, are in the poorest wealth category, live in rural areas, do not listen to radio, do not watch television, are currently employed, have never terminated a pregnancy, and have birth intervals between 24 and 36 months.

### 3.3. Pattern of Changes/Shift in Childbirth Location by Countries

Table 2 shows the pattern of changes in childbirth location by country. The results show that the changing pattern varies across the countries. The highest change is observed in Uganda where 20.36% (95% CI; 19.07–21.66) changed their location of childbirth, and the lowest is observed in Burkina Faso where only 5.24% (95% CI; 2.99–7.48) changed their location of childbirth. Furthermore, the highest shift from home to a healthcare facility is observed in Malawi with 62.33% (95% CI; 56.82–67.85), and the lowest shift from home to a healthcare facility is observed in Chad with 6.56% (95% CI; 5.80–7.31). We have included forest plots summarizing cross‐country differences in the percentage of women who changed childbirth location and the percentage shifting from home to a facility in Figures S1 and S2, respectively.

**Table 2:** Pattern of changes in childbirth location by countries.

Country (year of survey)	% Changing (95% CI)	% Changed from *H* to *F*
Angola (2015–2016)	16.32 (15.10–17.54)	13.83 (12.36–15.30)
Burkina Faso (2021)	5.24 (2.99–7.48)	31.58 (8.56–54.60)
Benin (2017–2018)	8.51 (7.58–9.44)	28.84 (25.16–32.52)
Burundi (2016–2017)	11.88 (10.76–13.00)	46.91 (42.21–51.61)
Côte d′Ivoire (2021)	15.18 (11.71–18.65)	26.44 (16.98–35.89)
Cameroon (2018)	13.29 (11.89–14.70)	18.61 (15.84–21.38)
Ethiopia (2016)	16.34 (14.86–17.83)	15.84 (14.19–17.49)
Gabon (2019–2021)	9.22 (7.65–10.78)	31.94 (24.24–39.65)
Ghana (2022)	12.14 (8.83–15.44)	32.14 (19.52–44.76)
Gambia (2019–2020)	19.17 (17.44–20.90)	48.25 (43.78–52.71)
Guinea (2018)	17.30 (15.61–19.00)	17.96 (15.63–20.28)
Kenya (2022)	14.46 (12.03–16.89)	30.05 (23.35–36.76)
Liberia (2019–2020)	19.82 (17.45–22.18)	48.20 (42.56–53.84)
Lesotho (2023–2024)	18.46 (8.77–28.15)	42.86 (‐6.58–92.29)
Madagascar (2021)	17.00 (15.47–18.52)	11.77 (10.17–13.37)
Mali (2018)	15.14 (13.76–16.53)	21.19 (18.62–23.75)
Mauritania (2019–2021)	12.76 (11.52 ‐ 14.00)	20.84 (18.08–23.60)
Malawi (2015–2016)	9.12 (8.13–10.12)	62.33 (56.82–67.85)
Mozambique (2022–2023)	12.35 (8.79–15.91)	17.48 (10.02–24.93)
Nigeria (2018)	14.07 (13.34–14.81)	10.87 (10.05–11.69)
Rwanda (2019–2020)	6.62 (5.38–7.85)	39.02 (28.24–49.81)
Sierra Leone (2019)	12.35 (10.91–13.79)	37.56 (32.81–42.32)
Senegal (2019)	6.08 (3.60–8.55)	33.33 (14.33–52.34)
Chad (2014–2015)	8.62 (7.84–9.41)	6.56 (5.80–7.31)
Togo (2013–14)	12.12 (10.50–13.75)	16.75 (13.72–19.79)
Tanzania (2022)	16.63 (13.25–20.01)	34.21 (23.30–45.12)
Uganda (2016)	20.36 (19.07–21.66)	36.42 (33.61–39.22)
South Africa (2016)	6.90 (4.55–9.26)	47.37 (22.64–72.09)
Zambia (2018)	16.56 (14.96–18.16)	46.06 (41.35–50.78)
Zimbabwe (2015)	17.29 (14.97–19.60)	45.17 (39.07–51.27)
**Total**	13.59 (13.31–13.86)	18.42 (17.94–18.91)

*Note:* 95% confidence interval (CI) values are in parentheses.

Abbreviations: F, facility; H, home.

### 3.4. Factors Associated With Changes in the Location of Childbirth

Table 3 presents the findings from the multivariable regression analyses of the factors associated with changes in childbirth location among SSA women of reproductive age. The results in Model 1 show the factors associated with changing the location of childbirth, and the results in Model 2 show the factors associated with switching the location of childbirth from home to a health facility. The unadjusted odds ratios are reported in Table S3.

**Table 3:** Factors associated with changes in the location of childbirth among sub‐Saharan African women of reproductive age.

	Changed birth location	Changed from home to facility
**Variables**	**Model 1—AOR (95% CI)**	**Model 2—AOR (95% CI)**
**Age**		
15–19	Ref	Ref
20–24	1.01 (0.88–1.16)	1.20 (0.94–1.52)
25–29	0.92 (0.80–1.06)	1.15 (0.91–1.46)
30–34	0.88 (0.76–1.02)	1.12 (0.88–1.44)
35–39	0.88 (0.75–1.03)	1.22 (0.95–1.57)
40–44	0.85 (0.71–1.02)	1.03 (0.78–1.37)
45–49	0.90 (0.68–1.20)	1.00 (0.68–1.48)

**Education level**		
No education	Ref	Ref
Primary	1.13 (1.05–1.22) ^∗∗^	1.26 (1.13–1.41) ^∗∗∗^
Secondary	1.01 (0.91–1.11)	1.87 (1.60–2.18) ^∗∗∗^
Higher	0.49 (0.37–0.65) ^∗∗∗^	2.76 (1.53–4.97) ^∗∗∗^

**Marital status**		
Married	Ref	Ref
Never in union	0.86 (0.73–1.02)	0.99 (0.74–1.34)
Living with a partner	0.90 (0.76–1.07)	0.91 (0.67–1.23)
Widowed	0.99 (0.73–1.36)	0.97 (0.60–1.57)
Divorced	0.79 (0.59–1.07)	0.55 (0.32–0.96) ^∗^
Separated	1.00 (0.80–1.25)	0.98 (0.67–1.45)

**Wealth**		
Poorest	Ref	Ref
Poorer	0.98 (0.91–1.06)	1.30 (1.17–1.45) ^∗∗∗^
Middle	1.02 (0.94–1.12)	1.52 (1.33–1.74) ^∗∗∗^
Richer	0.87 (0.78–0.96) ^∗∗^	1.78 (1.51–2.11) ^∗∗∗^
Richest	0.58 (0.50–0.68) ^∗∗∗^	2.31 (1.79–2.97) ^∗∗∗^

**Residence**		
Urban	Ref	Ref
Rural	1.00 (0.91–1.10)	0.66 (0.57–0.76) ^∗∗∗^

**Frequency of listening to radio**		
Not at all	Ref	Ref
Less than once a week	1.14 (1.05–1.23) ^∗∗∗^	1.19 (1.05–1.35) ^∗∗^
At least once a week	1.12 (1.03–1.21) ^∗∗^	1.28 (1.13–1.45) ^∗∗∗^
Almost every day	1.14 (0.88–1.48)	1.00 (0.69–1.46)

**Frequency of watching television**		
Not at all	Ref	Ref
Less than once a week	1.08 (0.98–1.19)	1.45 (1.24–1.68) ^∗∗∗^
At least once a week	0.90 (0.81–1.01)	1.35 (1.13–1.61) ^∗∗∗^
Almost every day	1.03 (0.81–1.31)	1.54 (1.04–2.29) ^∗^

**Currently working**		
No	Ref	Ref
Yes	1.00 (0.93–1.07)	1.07 (0.97–1.18)

**Ever terminated a pregnancy**		
No	Ref	Ref
Yes	1.11 (1.02–1.20) ^∗^	1.10 (0.97–1.26)

**Birth interval (months)**		
< 24	Ref	Ref
24–36	1.21 (1.12–1.30) ^∗∗∗^	1.34 (1.20–1.49) ^∗∗∗^
> 36	1.22 (1.12–1.33) ^∗∗∗^	1.61 (1.43–1.82) ^∗∗∗^

**Country fixed effect**	Yes	Yes
Constant	0.20 (0.16–0.25) ^∗∗∗^	0.07 (0.05–0.10) ^∗∗∗^
Observations	61,240	24,538

*Note:* 95% confidence interval (CI) values are in parentheses. Sampling weights and clustering were accounted for in all estimations.

Abbreviations: AOR, adjusted odds ratio; Ref, reference group.

^∗^ *p* < 0.05,  ^∗∗^ *p* < 0.01,  ^∗∗∗^ *p* < 0.001.

The results from Model 1 revealed that women with primary education are 13% more likely to change birth location compared with women with no education (AOR = 1.13, 95% CI: 1.05–1.22). However, women with higher education are 51% less likely to change birth location compared with women with no education (AOR = 0.49, 95% CI: 0.37–0.65). Women in the richer (AOR = 0.87, 95% CI: 0.78–0.96) and richest (AOR = 0.58, 95% CI: 0.50–0.68) wealth categories are less likely to change birth location compared with those in the poorest wealth category. Furthermore, women who listen to the radio less than once a week (AOR = 1.14, 95% CI: 1.05–1.23) and those who listen to the radio at least once a week (AOR = 1.12, 95% CI: 1.03–1.21) are more likely to switch birth location compared with those who do not listen at all. Also, the likelihood of changing birth location is higher for women who have ever terminated a pregnancy than those who have not (AOR = 1.11, 95% CI: 1.02–1.20). Moreover, the odds of changing birth location increase as birth interval (in months) increases, with women with a birth interval of 36 months plus being more likely to change birth location (AOR = 1.22, 95% CI: 1.12–1.33) compared with those with a birth interval of less than 24 months.

The results from Model 2 revealed that the probability of changing birth location from home to a healthcare facility increases with the level of education, with those with higher education having a higher likelihood of changing from home to a facility (AOR = 2.76, 95% CI: 1.53–4.97) compared with those with no education. Furthermore, the odds of changing birth location from home to a healthcare facility increase with wealth status, particularly for women in the richest category (AOR = 2.31, 95% CI: 1.79–2.97) relative to those in the poorest category. Women in rural areas are 34% less likely to change from home to a facility compared with those in urban areas (AOR = 0.66, 95% CI: 0.57–0.76). Also, women who listen to the radio less than once a week (AOR = 1.19, 95% CI: 1.05–1.35) and those who listen to the radio at least once a week (AOR = 1.28, 95% CI: 1.13–1.45) are more likely to change birth location from home to a facility compared with those who do not listen at all. Likewise, the probability of changing birth location from home to a healthcare facility increases with the frequency of watching television, and this was higher for women who watch television almost every day (AOR = 1.54, 95% CI: 1.04–2.29) compared with those who do not watch at all. Moreover, the odds of changing birth location from home to a healthcare facility increase as the birth interval (in months) increases, with women with a birth interval of 36 months plus being more likely to change from home to facility (AOR = 1.61, 95% CI: 1.43–1.82) compared with those with a birth interval of less than 24 months.

## 4. Discussion

In this population‐based study utilizing the most recent DHS data of 30 SSA countries, we examined the prevalence and determinants of changes in childbirth location among women of reproductive age. The overall prevalence of women changing their childbirth location was 13.6%, with a majority (54.4%) transitioning from home to healthcare facilities. The highest prevalence of change in childbirth location was recorded in Uganda (20.4%), whereas the lowest was in Burkina Faso (5.2%). The determinants of changes in childbirth location included country, education, wealth status, residence, media exposure (radio and television), and birth intervals.

Our study revealed stark cross‐country variations in changes in childbirth location, which may be driven by distinct healthcare investments, cultural norms, and structural barriers. These disparities highlight the uneven progress in transitioning from home to healthcare facilities across SSA. Many countries in East and Southern Africa recorded higher prevalence rates of transitioning to healthcare facilities. For instance, Malawi recorded the highest shift from home to healthcare facilities (62.3%), followed by Zimbabwe (45.2%).

These findings are consistent with previous literature in these countries, reflecting the impact of targeted maternal health policies (e.g., development of maternal and perinatal death surveillance and response systems) and stronger healthcare systems in these regions that reduce financial and geographic barriers to institutional deliveries [25, 29, 30]. On the other hand, countries like Chad (8.6%) and Burkina Faso (5.2%) reported much lower rates of change. This is not surprising for Chad, as persistent socioeconomic challenges, limited healthcare infrastructure, and cultural norms favoring home births have been consistently reported in the literature as significant barriers to progress. [8, 31]. Similarly, Burkina Faso′s low prevalence can be attributed to political instability and security challenges, which have disrupted healthcare systems and service delivery.

Over the past decade, the country has experienced multiple coup d′états, increasing violence from extremist groups, and widespread internal displacement. These factors have resulted in the closure of healthcare facilities, reduced investment in maternal health programs, and a reinforcement of cultural norms favoring home births [32–34]. Women in displaced populations also face significant barriers to accessing healthcare facilities, further contributing to the low rates of change. An important intervention common in countries with high transitions to healthcare facilities, such as Malawi, is the implementation of community‐based mobilization programs [8, 30, 35]. In Malawi, facilitators lead health‐focused discussion groups that improve knowledge about maternal health and encourage facility‐based deliveries [36, 37]. Similarly, Zambia has implemented safe motherhood action groups (SMAGs) that engage community members to promote institutional childbirth [35, 38]. These interventions highlight the success of demand‐side strategies in improving healthcare utilization, and countries with a low prevalence of transitions, such as Chad and Burkina Faso, could adopt these models.

Wealth emerged as a critical determinant of changes in childbirth location. Unlike contexts [30, 39, 40] where extensive subsidies, such as fee exemptions and insurance packages, minimize the effect of financial status, this study revealed that women in the richest group were significantly more likely to seek institutional care (AOR = 2.31, 95% CI: 1.79–2.97). This finding highlights the persistent financial barriers that prevent economically disadvantaged women in SSA from accessing institutional care, as also noted by [41, 42]. The revelation of wealth impacting the decision of changing birth locations is not surprising in this subregion, especially considering that SSA region is among the regions in the world plagued with inequalities, with wide financial segregation; a financial status of being wealthy may translate to a woman′s ability to afford both direct and indirect costs of childbirth. This may include, but is not limited to, out‐of‐pocket payments, transport fares, informal charges, and unexpected costs such as emergency cesarean section surgeries. In most SSA countries, where healthcare systems operate on a pay‐as‐you‐go model, having wealth goes a long way toward ensuring timely access to services during conception, the intrapartum period, and the postpartum period [43].

In addition to wealth, education displayed a similarly strong influence on childbirth location transitions. Women with higher education were more likely to transition from home to healthcare facilities (AOR: 2.76, 95% CI: 1.53–4.97) compared with those without formal education, corroborating previous work associating formal education with increased autonomy [44–47]. Education provides women with knowledge about the benefits of skilled birth attendance, empowering them to make informed health decisions. However, our study revealed a unique observation: Those with only primary education often changed their childbirth location without necessarily opting for healthcare facilities. This may indicate that although primary education raises awareness of the need for skilled care, it is insufficient to overcome other barriers such as financial constraints, cultural norms, or lack of nearby facilities. These findings highlight the need for educational interventions to be complemented by broader strategies, such as reducing costs with pro‐poor health interventions and improving access to healthcare facilities.

Adde et al.′s [8] study examining the prevalence and determinants of the place of delivery among reproductive‐age women in 28 SSA countries reported rural–urban disparities. Our findings support this and extend the literature by providing estimates for overall changes in birth locations and incorporating data from five additional SSA countries, including Angola, Burkina Faso, Zimbabwe, Madagascar, and Mauritania, as well as updated data. We found that rural women were less likely to transition to healthcare facilities compared with urban women (AOR = 0.66, 95% CI: 0.57–0.76). This is consistent with findings from other studies that highlight limited healthcare infrastructure and longer distances to health facilities in rural areas as key barriers [8, 19, 48]. Women in rural areas often face additional challenges such as poor road networks and high‐transportation costs, which deter them from seeking facility‐based care [19].

Globally, media exposure has been reported to influence health outcomes including maternal and child health outcomes. Our findings align with this global trend as we realized that media exposure played a significant role in influencing changes in childbirth location. Women who listened to the radio or watched television frequently were more likely to transition to healthcare facilities. This finding reflects the increasing importance of electronic media in shaping health‐seeking behaviors. Health‐related programming on radio and television serves as a vital source of information, improving knowledge of danger signs during pregnancy, enabling early identification of high‐risk pregnancies, and helping clear misconceptions that favor home deliveries, especially in areas with low literacy rates. We believe that repeated exposure to the same health message through media campaigns may go a long way toward shaping societal norms by normalizing facility‐based deliveries in regions and communities that frown upon them, as well as encouraging early preparation for transport and other delivery‐related costs, especially in communities where traditional home deliveries are the norm. As such, expanding media campaigns with targeted reproductive health content could further enhance maternal healthcare utilization.

A global study examining the impact of child spacing on maternal and perinatal outcomes across all United Nations subregions including all the regions in SSA found that shorter birth intervals are associated with increased risks of adverse maternal and perinatal outcomes, whereas longer intervals contribute to improved health outcomes for both mothers and newborns [49]. In alignment with these findings, our study revealed that longer birth intervals significantly increased the likelihood of transitioning from home to healthcare facilities, a critical modifiable factor in addressing adverse outcomes. Women with birth intervals exceeding 36 months were more likely to plan and access skilled care during subsequent deliveries. This may be attributed to the additional time available for antenatal care visits, heightened maternal awareness of health risks, and better preparedness to utilize healthcare services as stipulated by other literature on the importance of childbirth spacing. [50, 51].

We found that the odds of choosing a health facility as the place of delivery declined with age among reproductive‐age women in SSA. This finding is consistent with previous studies that explain younger women, particularly first‐time mothers, tend to seek health facility deliveries due to a heightened perception of risk associated with complications during childbirth [52, 53]. In contrast, older, multiparous women may perceive themselves as less susceptible to complications and rely more on traditional birth attendants (TBAs) or home deliveries, particularly in settings where TBAs are culturally favored for their perceived friendliness compared with SBAs [8, 53]. This trend was particularly pronounced among women in their last reproductive years (45–49), who recorded the highest prevalence of home births in our study, consistent with the findings of Adde et al. [8]. These observations highlight the need for targeted interventions to address the unique needs of older women, including community‐based awareness campaigns to emphasize the benefits of skilled care for all women regardless of parity or age.

Although we have identified several determinants of changes in childbirth location, we would like to emphasize that these relationships are associational, not causal, given the cross‐sectional nature of the DHS data. Although we cannot make causal inferences about these factors, our findings could be interpreted through the lens of Andersen′s Behavioral Model of Health Services Use [21, 22], which conceptualizes health service utilization as a function of predisposing characteristics, enabling resources and perceived or evaluated need. In our study, age and education represent predisposing characteristics that may influence health beliefs, knowledge, and risk perception regarding childbirth, whereas wealth, place of residence (i.e., rural vs. urban), and media exposure represent enabling resources that may influence whether women are able to access facility‐based delivery services. Birth spacing and women′s reproductive history (e.g., parity and adverse pregnancy outcomes) may approximate need through perceived vulnerability and readiness for skilled care in subsequent pregnancies. This framework contextualizes our findings better by suggesting that transitions in childbirth location across births are shaped by the interaction between readiness to seek care and the enabling conditions that make facility delivery feasible. It also supports interpretation of cross‐country variation, since countries differ in health system organization, geographic distribution of services, and broader conditions including insecurity and displacement that can constrain service availability and utilization. Additionally, the observed associations for wealth and rural residence further suggest that enabling constraints may be a key barrier to shifting toward facility delivery in many settings.

### 4.1. Strengths and Limitations

Our study should be interpreted in light of its strengths and limitations. It is among the few to provide a comprehensive assessment of the prevalence and determinants of childbirth locations in SSA, a region with a disproportionate burden of maternal and neonatal mortality. We extend the literature by providing overall estimates for changes in childbirth location, followed by an in‐depth assessment of transitions from home to healthcare facilities. This dual approach offers a more complete picture of how the relationship with various determinants unfolds. Additionally, we incorporate data from five additional SSA countries, Angola, Burkina Faso, Zimbabwe, Madagascar, and Mauritania along with updated data, further enhancing the robustness and relevance of our findings. The use of high‐quality, nationally representative data from 30 SSA countries from the DHS, known for their standardized collection methods and reliability, enhances the generalizability of our findings. Furthermore, the study identified novel findings, such as the tendency for women with primary education to change childbirth locations without necessarily opting for healthcare facilities, contributing new perspectives to the existing literature.

However, the study has several limitations. The cross‐sectional design limits the ability to infer causal relationships between determinants and changes in childbirth location. Additionally, we relied on self‐reported data which may introduce recall bias, particularly when women report on past childbirths. Although the study highlights key determinants, it does not fully explore context‐specific factors such as local healthcare policies, cultural practices, or differences in healthcare infrastructure, which could further explain the observed trends. Moreover, the analysis excluded potential determinants such as healthcare quality and the role of partners in decision‐making, which may also influence childbirth location. Also, although our analysis includes 30 SSA countries, generalizability across the subregions should be interpreted with caution because countries differ in survey timing and context, and pooled estimates may mask important within‐country and subnational differences. Nonetheless, the findings are likely to be relevant to other settings with similar demographic profiles and contextual conditions, including comparable health system constraints that influence access to facility‐based childbirth. Furthermore, we focused only on the two most recent births, which reduces the chances of recall error but may have overlooked longer‐term trends for women with more than two children. Lastly, supply‐side factors such as availability, accessibility, and quality of healthcare facilities, which are critical to understanding maternal healthcare utilization, were not included in the analysis due to the unavailable data in DHS. As such, future research should use longitudinal designs to examine how women′s childbirth location decisions change over time and incorporate qualitative and mixed methods to explore contextual influences and healthcare supply‐side factors, including access, facility readiness, and perceived quality of care to better understand and address the barriers to institutional childbirth in the region.

## 5. Conclusion and Recommendations

Our study highlights significant disparities in changes in childbirth locations across SSA countries, shaped by the combined influence of enabling resources and access conditions rather than a single factor. We observed that wealth, education, and living in rural areas are strongly linked to whether women transition to facility‐based delivery. This may suggest that a woman′s ability to transition to facility‐based childbirth may depend on her financial capabilities, health knowledge, and proximity to a facility, which differ greatly between countries. The role of media exposure and birth spacing further suggests that information and preparedness support transitions, but these gains are likely constrained where affordability and access barriers persist.

Although countries like Malawi and Zimbabwe demonstrate successful strategies for promoting facility‐based deliveries, others like Chad and Burkina Faso face persistent barriers, including political instability, weak healthcare systems, and sociocultural challenges. Because of these cross‐country variations, recommendations should be tailored to specific countries and/or regions within SSA. Specifically, in East and Southern African countries (e.g., Zimbabwe and Malawi) where higher transitions were observed, strengthening and scaling up proven demand side interventions such as community‐based mobilization, alongside continued health system strengthening, may yield additional gains, particularly among rural and poorer women. In fragile contexts, particularly in parts of West Africa (e.g., Burkina Faso) where insecurity and displacement undermine service delivery, priorities should include restoring and protecting essential maternal health services, strengthening community‐level outreach, and adopting adaptive delivery approaches, such as mobile outreach services and integrating maternal care into humanitarian response. In the broader SSA context, we advocate for effective pro‐poor financing strategies, such as fee exemptions and equitable insurance coverage, that reduce cost barriers disproportionately affecting the poorest women while ensuring implementation reaches those most in need. Altogether, addressing these disparities requires targeted interventions, including expanding rural healthcare infrastructure, promoting female education, leveraging mass media for health awareness, and implementing pro‐poor healthcare policies. These efforts are essential for achieving equitable maternal healthcare access and meeting global targets, particularly, SDG 3.1, aimed at reducing maternal mortality to fewer than 70 deaths per 100,000 live births.

## Author Contributions

S.Y. conceived the study; A.B. conducted the analysis and drafted the methods; S.Y., A.B., G.B., and M.S. contributed to the development of the manuscript and reviewed drafts to ensure important intellectual content.

## Funding

No funding was received for this manuscript.

## Disclosure

S.Y. had the final responsibility to submit. All authors have read and approved the final version for submission.

## Ethics Statement

We used a secondary dataset that is freely available to the public from the DHS Program; therefore, no ethical approval was requested. The dataset is anonymized and more details regarding its ethical standards are available at http://goo.gl/ny8T6X.

## Consent

The authors have nothing to report.

## Conflicts of Interest

The authors declare no conflicts of interest.

## Supporting information


**Supporting Information** Additional supporting information can be found online in the Supporting Information section. The supporting information provides additional statistical details and visualizations supporting the study′s findings. Table S1: The diagnostic tests for multicollinearity, including variance inflation factors (VIFs) and tolerance indices (TI) for all independent variables included in the regression models. Table S2: The demographic and socioeconomic characteristics of the specific subset of women who switched from facility‐based delivery to home delivery. Table S3: Reports on the unadjusted crude odds ratios (COR) with 95% confidence intervals for factors associated with changes in childbirth location. Figure S1: A forest plot summarizing the percentage of women who changed childbirth location across the 30 included countries, illustrating the variations in prevalence. Figure S2: A forest plot focusing specifically on the percentage of women who shifted from home delivery to a healthcare facility, highlighting country‐specific shifts in health‐seeking behavior.

## Data Availability

The dataset used for this study is available in a public, open access repository. The dataset can be accessed via https://dhsprogram.com/data/available-datasets.cfm.

## References

[1] World Health Organization , Maternal Mortality, 2025, World Health Organization, accessed January 28, 2025 https://www.who.int/news-room/fact-sheets/detail/maternal-mortality.

[2] Amu H. and Nyarko S. H. , Preparedness of Health Care Professionals in Preventing Maternal Mortality at a Public Health Facility in Ghana: a Qualitative Study, BMC Health Services Research. (2016) 16, no. 1, 10.1186/s12913-016-1527-y, 2-s2.0-84978251008, 27405375. PMC4942930 27405375

[3] World Health Organization , Transforming our world: the 2030 Agenda for Sustainable Development, 2015, Department of Economic and Social Affairs, accessed January 28, 2025 https://sdgs.un.org/2030agenda.

[4] World Health Organization , Ending Preventable Maternal Mortality (EPMM): A Renewed Focus for Improving Maternal and Newborn Health and Well-Being, 2021, World Health Organization, accessed January 28, 2025 https://www.who.int/publications/i/item/9789240040519.

[5] Musarandega R. , Nyakura M. , Machekano R. , Pattinson R. , and Munjanja S. P. , Causes of Maternal Mortality in Sub-Saharan Africa: A Systematic Review of Studies Published from 2015 to 2020, Journal of Global Health. (2021) 11, 04048, 10.7189/jogh.11.04048, 34737857. 34737857 PMC8542378

[6] Ameyaw E. K. , Baatiema L. , Naawa A. , Odame F. , Koramah D. , Arthur-Holmes F. , Frimpong S. O. , and Hategeka C. , Quality of Antenatal Care in 13 Sub-Saharan African countries in the SDG Era: Evidence From Demographic and Health Surveys, BMC Pregnancy Childbirth. (2024) 24, no. 1, 10.1186/s12884-024-06459-2, 38654217. PMC11040817 38654217

[7] Chinkhumba J. , De Allegri M. , Muula A. S. , and Robberstad B. , Maternal and Perinatal Mortality by Place of Delivery in Sub-Saharan Africa: A Meta-Analysis of Population-Based Cohort Studies, BMC Public Health. (2014) 14, no. 1, 10.1186/1471-2458-14-1014, 2-s2.0-84908003332, 25263746. PMC4194414 25263746

[8] Adde K. S. , Dickson K. S. , and Amu H. , Prevalence and Determinants of the Place of Delivery Among Reproductive Age Women in Sub–Saharan Africa, PLoS ONE. (2020) 15, no. 12, e0244875, 10.1371/journal.pone.0244875, 33382825. 33382825 PMC7774912

[9] Onambele L. , Ortega-Leon W. , Guillen-Aguinaga S. , Forjaz M. J. , Yoseph A. , Guillen-Aguinaga L. , Alas-Brun R. , Arnedo-Pena A. , Aguinaga-Ontoso I. , and Guillen-Grima F. , Maternal Mortality in Africa: Regional Trends (2000-2017), International Journal of Environmental Research and Public Health. (2022) 19, no. 20, 13146, 10.3390/ijerph192013146, 36293727. 36293727 PMC9602585

[10] Bomela N. J. , Maternal Mortality by Socio-Demographic Characteristics and Cause of Death in South Africa: 2007–2015, BMC Public Health. (2020) 20, no. 1, 10.1186/s12889-020-8179-x, 32007100. PMC6995651 32007100

[11] Gebreegziabher S. B. , Marrye S. S. , Kumssa T. H. , Merga K. H. , Feleke A. K. , Dare D. J. , Hallström I. K. , Yimer S. A. , and Shargie M. B. , Assessment of Maternal and Child Health Care Services Performance in the Context of COVID-19 Pandemic in Addis Ababa, Ethiopia: Evidence From Routine Service Data, Reproductive Health. (2022) 19, no. 1, 10.1186/s12978-022-01353-6, 35164776. PMC8842853 35164776

[12] Ossai E. N. , Eze I. I. , Eke P. C. , Onah C. K. , Agu C. , and Ogbonnaya L. U. , Where, Why and Who Delivers Our Babies? Examining the Perspectives of Women on Utilization of Antenatal and Delivery Services in a Developing Country, BMC Pregnancy Childbirth. (2023) 23, no. 1, 10.1186/s12884-022-05306-6, 36593447. PMC9806875 36593447

[13] Olubodun T. , Ogundele O. A. , Michael T. O. , Okunlola O. A. , Olubodun A. B. , and Rahman S. A. , Regional Trends, Spatial Patterns and Determinants of Health Facility Delivery Among Women of Reproductive Age in Nigeria: A National Population Based Cross-Sectional Study, PLOS ONE. (2024) 19, e0312005, 10.1371/journal.pone.0312005. 39413110 PMC11482673

[14] UNICEF DATA , Delivery care, 2024, UNICEF DATA, (accessed January 28, 2025). https://data.unicef.org/topic/maternal-health/delivery-care/.

[15] Montagu D. , Sudhinaraset M. , Diamond-Smith N. , Campbell O. , Gabrysch S. , Freedman L. , Kruk M. E. , and Donnay F. , Where Women Go to Deliver: Understanding the Changing Landscape of Childbirth in Africa and Asia, Health Policy Plan. (2017) 32, no. 8, 1146–1152, 10.1093/heapol/czx060, 2-s2.0-85030673609, 28541422. 28541422 PMC5886217

[16] Straneo M. , Hanson C. , van den Akker T. , Afolabi B. B. , Asefa A. , Delamou A. , Dennis M. , Gadama L. , Mahachi N. , Mlilo W. , Pembe A. B. , Tsuala Fouogue J. , and Beňová L. , Inequalities in Use of Hospitals for Childbirth Among Rural Women in Sub-Saharan Africa: A Comparative Analysis of 18 Countries Using Demographic and Health Survey data, BMJ Global Health. (2024) 9, no. 1, 10.1136/bmjgh-2023-013029, 38262683. PMC10806834 38262683

[17] Peer N. , The Converging Burdens of Infectious and Non-Communicable Diseases in Rural-to-Urban Migrant Sub-Saharan African Populations: A Focus on HIV/AIDS, Tuberculosis and Cardio-Metabolic Diseases, Tropical Diseases, Travel Medicine and Vaccines. (2015) 1, no. 1, 10.1186/s40794-015-0007-4, 28883938. PMC5526364 28883938

[18] Dankwah E. , Zeng W. , Feng C. , Kirychuk S. , and Farag M. , The social Determinants of Health Facility Delivery in Ghana, Reproductive Health. (2019) 16, no. 1, 10.1186/s12978-019-0753-2, 2-s2.0-85068826701, 31291958. PMC6617857 31291958

[19] Kifle M. M. , Kesete H. F. , Gaim H. T. , Angosom G. S. , and Araya M. B. , Health Facility or Home Delivery? Factors Influencing the Choice of Delivery Place Among Mothers Living in Rural Communities of Eritrea, Journal of Health, Population and Nutrition. (2018) 37, no. 1, 10.1186/s41043-018-0153-1, 2-s2.0-85055202440, 30348219. PMC6196428 30348219

[20] Ngowi A. F. , Kamazima S. R. , Kibusi S. , Gesase A. , and Bali T. , Women′s Determinant Factors for Preferred Place of Delivery in Dodoma Region Tanzania: A Cross Sectional Study, Reproductive Health. (2017) 14, no. 1, 10.1186/s12978-017-0373-7, 2-s2.0-85028917707, 28877749. PMC5588730 28877749

[21] Andersen R. M. , Revisiting the Behavioral Model and Access to Medical Care: Does it Matter?, Journal Of Health and Social Behavior. (1995) 36, no. 1, 1–10, 10.2307/2137284, 2-s2.0-0029270196, 7738325. 7738325

[22] Andersen R. and Newman J. F. , Societal and Individual Determinants of Medical Care Utilization in the United States, Milbank Quarterly. (2005) 83, no. 4, 10.1111/j.1468-0009.2005.00428.x, 2-s2.0-84937074079. 4198894

[23] Corsi D. J. , Neuman M. , Finlay J. E. , and Subramanian S. V. , Demographic and Health Surveys: A Profile, International Journal of Epidemiology. (2012) 41, no. 6, 1602–1613, 10.1093/ije/dys184, 2-s2.0-84872122723. 23148108

[24] Aliaga A. and Ren R. , Cluster Optimal Sample Size for Demographic and Health Surveys, 7th International Conference on Teaching Statistics–ICOTS. (2006) 7, 1–6.

[25] Dehury B. and Chourase M. , Does the Preference for Location of Childbirth Change for Successive Births? Evidence From the States and Regions of India, Journal of Biosocial Science. (2021) 53, no. 2, 266–289, 10.1017/S0021932020000188, 32295667. 32295667

[26] Dixit P. and Dwivedi L. K. , Utilization of Institutional Delivery Services Across Successive Births in India, International Journal of Population Studies. (2016) 2, no. 2, 10.18063/ijps.2016.02.006.

[27] Long J. S. and Freese J. , Regression Models for Categorical Dependent Variables Using Stata, 2006, Stata Press.

[28] Menard S. , Applied Logistic Regression Analysis, 2001, 2nd edition, Sage Publications.

[29] Musarandega R. , Ngwenya S. , Murewanhema G. , Machekano R. , Magwali T. , Nystrom L. , Pattinson R. , Munjanja S. , the Zimbabwe Maternal and Perinatal Mortality Study Group , Chikutiro A. , Mahomva A. , Mangombe A. , Madzima B. , Guzha B. , Chimamise C. , Gwanzura C. , Makosa D. , Ziki E. , Ngaru E. , Tahuringana E. , Madziyire G. , Murewanhema G. , Chimhini G. , Kasule J. , Chirengwa J. , Gondongwe L. , Nyandoro M. , Chirehwa M. , Parirenyatwa M. M. , Gaza M. , Nyakura M. , Gona N. , Musarandega R. , Mataya R. , Makoni R. , Gunguwo S. , Magwali T. , Magure T. , Mushangwe V. , Dondo V. , and Chirombe W. , Changes in Causes of Pregnancy-Related and Maternal Mortality in Zimbabwe 2007–08 to 2018–19: Findings From Two Reproductive Age Mortality Surveys, BMC Public Health. (2022) 22, no. 1, 10.1186/s12889-022-13321-7, 35534811. PMC9087911 35534811

[30] Allegri M. D. , Chase R. P. , Lohmann J. , Schoeps A. , Muula A. S. , and Brenner S. , Effect of Results-Based Financing on Facility-Based Maternal Mortality at Birth: An Interrupted Time-Series Analysis With Independent Controls in Malawi, Health. (2019) 4, no. 3, e001184, 10.1136/bmjgh-2018-001184, 2-s2.0-85068185529, 31297244. PMC6590974 31297244

[31] Kim S. and Kim S.-Y. , Exploring Factors Associated With Maternal Health Care Utilization in Chad, Journal of Global Health Science. (2019) 1, e31, 10.35500/jghs.2019.1.e31.

[32] Faso B. , U S Dep State, 2022, accessed January 28, 2025 https://www.state.gov/reports/2022-country-reports-on-human-rights-practices/burkina-faso/.

[33] Faso B. , When Insecurity, Conflict and Other Challenges Get Between People and the Healthcare, the REACH Initiative Connects People With the Care They Need, 2024, accessed January 28, 2025 https://www.ifrc.org/article/burkina-faso-when-insecurity-conflict-and-other-challenges-get-between-people-and.

[34] Ukoaka B. M. , Daniel F. M. , Ajah K. U. , Abiodun A. , Udam N. G. , Daniel R. T. , Udoh R. A. , Lawal H. , Ogunbowale I. , and Olalekan O. J. , Exploring the Socioeconomic and Political Implications of Recurrent Coup d′etat in Africa and Their Impact on Global Health, Discover Global Society. (2024) 2, no. 1, 10.1007/s44282-024-00077-1.

[35] Hurst T. E. , Semrau K. , Patna M. , Gawande A. , and Hirschhorn L. R. , Demand-Side Interventions for Maternal Care: Evidence of More use, not better outcomes, Not Better Outcomes. BMC Pregnancy Childbirth. (2015) 15, no. 1, 10.1186/s12884-015-0727-5, 2-s2.0-84947485066, 26566812. PMC4644345 26566812

[36] Kim E. T. , Singh K. , Speizer I. S. , Angeles G. , and Weiss W. , Availability of Health Facilities and Utilization of Maternal and Newborn Postnatal Care in RURAL MALAWI, BMC Pregnancy Childbirth. (2019) 19, no. 1, 10.1186/s12884-019-2534-x, 31847872. PMC6918704 31847872

[37] Walsh A. , Matthews A. , Manda-Taylor L. , Brugha R. , Mwale D. , Phiri T. , and Byrne E. , The Role of the Traditional Leader in Implementing Maternal, Newborn and Child Health Policy in Malawi, Health Policy Plan. (2018) 33, no. 8, 879–887, 10.1093/heapol/czy059, 2-s2.0-85054430393, 30084938. 30084938

[38] Johns B. , Ramchandani N. , Comfort A. , and Chankova S. , Impact of Safe Motherhood Action Groups on Use of Maternal Health Care in Zambia, 2014, Zambia Integrated Systems Strengthening Project, Abt Associates Inc.

[39] Azaare J. , Akweongo P. , Aryeetey G. C. , and Dwomoh D. , Impact of Free Maternal Health Care Policy on Maternal Health Care Utilization and Perinatal Mortality in Ghana: Protocol Design for Historical Cohort Study, Reproductive Health. (2020) 17, no. 1, 10.1186/s12978-020-01011-9, 33126906. PMC7597017 33126906

[40] Hatt L. E. , Makinen M. , Madhavan S. , and Conlon C. M. , Effects of User Fee Exemptions on the Provision and Use of Maternal Health Services: A Review of Literature, Journal of Health, Population, and Nutrition. (2013) 31, S67–S80.24992804

[41] Micah A. E. and Hotchkiss D. R. , Community-Level Factors Associated With the Use of Facility-Based Delivery Assistance in Uganda: A Multilevel Analysis, BMC Pregnancy Childbirth. (2020) 20, no. 1, 10.1186/s12884-020-2851-0, 32245431. PMC7118861 32245431

[42] Mwaliko E. , Downing R. , O’Meara W. , Chelagat D. , Obala A. , Downing T. , Simiyu C. , Odhiambo D. , Ayuo P. , Menya D. , and Khwa-Otsyula B. , Not Too Far to Walk the Influence of Distance on Place of Delivery in a Western Kenya Health Demographic Surveillance System, BMC Health Services Research. (2014) 14, no. 1, 10.1186/1472-6963-14-212, 2-s2.0-84901695101, 24884489. PMC4036729 24884489

[43] Asefa A. , Gebremedhin S. , Marthias T. , Nababan H. , Christou A. , Semaan A. , Banke-Thomas A. , Tabana H. , al-beity F. M. A. , Dossou J. P. , Gutema K. , Delvaux T. , Birabwa C. , Dennis M. , Grovogui F. M. , McPake B. , and Beňová L. , Wealth-Based Inequality in the Continuum of Maternal Health Service Utilisation in 16 sub-Saharan African Countries, International Journal for Equity in Health. (2023) 22, no. 1, 10.1186/s12939-023-02015-0, 37784140. PMC10544383 37784140

[44] Karlsen S. , Say L. , Souza J.-P. , Hogue C. J. , Calles D. L. , Gülmezoglu A. M. , and Raine R. , The Relationship Between Maternal Education and Mortality Among Women Giving Birth in Health Care Institutions: Analysis of the Cross Sectional WHO Global Survey on Maternal and Perinatal Health, BMC Public Health. (2011) 11, no. 1, 10.1186/1471-2458-11-606, 2-s2.0-79960883661, 21801399. PMC3162526 21801399

[45] Kibria G. M. A. , Albrecht J. , Lane W. , Stafford K. A. , Jones L. , Vesselinov R. , and Hirshon J. M. , Prevalence, Trends, and Factors Associated With Maternal Autonomy Regarding Healthcare, Finances, and Mobility in Bangladesh: Analysis of Demographic and Health Surveys 1999–2018, PLOS Global Public Health. (2024) 4, no. 2, e0002816, 10.1371/journal.pgph.0002816, 38306319. 38306319 PMC10836669

[46] Mensch B. S. , Chuang E. K. , Melnikas A. J. , and Psaki S. R. , Evidence for Causal Links Between Education and Maternal and Child Health: Systematic Review, Tropical Medicine & International Health. (2019) 24, no. 5, 504–522, 10.1111/tmi.13218, 2-s2.0-85063592931, 30767343. 30767343 PMC6519047

[47] Teshale A. B. , Biney G. K. , Sarfo M. , Ameyaw E. K. , and Yaya S. , What Do Mothers Know About Nutrition? Impacts on Childhood Nutrition Outcomes in Sub-Saharan Africa, Maternal and Child Health Journal. (2025) 29, no. 3, 349–360, 10.1007/s10995-025-04052-3, 39804446. 39804446

[48] Yaya S. , Uthman O. A. , Okonofua F. , and Bishwajit G. , Decomposing the Rural-Urban Gap in the Factors of Under-Five Mortality in Sub-Saharan Africa? Evidence From 35 countries, BMC Public Health. (2019) 19, no. 1, 10.1186/s12889-019-6940-9, 2-s2.0-85066414161, 31113395. PMC6528236 31113395

[49] Molitoris J. , Barclay K. , and Kolk M. , When and Where Birth Spacing Matters for Child Survival: An International Comparison Using the DHS, Demography. (2019) 56, no. 4, 1349–1370, 10.1007/s13524-019-00798-y, 2-s2.0-85068866537, 31270780. 31270780 PMC6667399

[50] Asif M. F. , Ishtiaq S. , Abbasi N. I. , Tahir I. , Abid G. , and Lassi Z. S. , The Interaction Effect of Birth Spacing and Maternal Healthcare Services on Child Mortality in Pakistan, Children. (2023) 10, no. 4, 10.3390/children10040710, 37189963. PMC10136845 37189963

[51] Lateef M. A. , Kuupiel D. , Mchunu G. G. , and Pillay J. D. , Utilization of Antenatal Care and Skilled Birth Delivery Services in Sub-Saharan Africa: A Systematic Scoping Review, International Journal of Environmental Research and Public Health. (2024) 21, no. 4, 10.3390/ijerph21040440, 38673351. PMC11050659 38673351

[52] Ntambue A. M. , Malonga F. K. , Dramaix-Wilmet M. , and Donnen P. , Determinants of Maternal Health Services Utilization in Urban Settings of the Democratic Republic of Congo—A Case study of Lubumbashi City, BMC Pregnancy Childbirth. (2012) 12, 10.1186/1471-2393-12-66, 2-s2.0-84866521080, 22780957. PMC3449182 22780957

[53] Onta S. , Choulagai B. , Shrestha B. , Subedi N. , Bhandari G. P. , and Krettek A. , Perceptions of Users and Providers on Barriers to Utilizing Skilled Birth Care in Mid- and Far-Western Nepal: A Qualitative Study, Global Health Action. (2014) 7, no. 1, 24580, 10.3402/gha.v7.24580, 2-s2.0-84928754483, 25119066. 25119066 PMC4131000

