# Supplementary material for: Where Women Give Birth Is Changing in Sub‐Saharan Africa: Evidence From 30 Countries Using DHS Data
Source: J Pregnancy. 2026 Mar 23;2026:2785076. doi: 10.1155/jp/2785076 (PMC13140859; doi:10.1155/jp/2785076)
Supplement: Supplementary file 1 — Supporting Information Additional supporting information can be found online in the Supporting Information section. The supporting information provides additional statistical details and visualizations supporting the study′s findings. Table S1: The diagnostic tests for multicollinearity, including variance inflation factors (VIFs) and tolerance indices (TI) for all independent variables included in the regression models. Table S2: The demographic and socioeconomic characteristics of the specific subset of women who switched from facility‐based delivery to home delivery. Table S3: Reports on the unadjusted crude odds ratios (COR) with 95% confidence intervals for factors associated with changes in childbirth location. Figure S1: A forest plot summarizing the percentage of women who changed childbirth location across the 30 included countries, illustrating the variations in prevalence. Figure S2: A forest plot focusing specifically on the percentage of women who shifted from home delivery to a healthcare facility, highlighting country‐specific shifts in health‐seeking behavior. [file JP-2026-2785076-s001.docx]

# **APPENDIX**

**Table A1.** Variance Inflation Factor (VIF) and Tolerance Indices (TI)

| **Variable** | **Variance Inflation Factor (VIF)** | **Tolerance Indices (TI)** |
| --- | --- | --- |
| Age | 1.05 | 0.95 |
| Education level | 1.33 | 0.75 |
| Marital status | 1.02 | 0.98 |
| Wealth | 1.57 | 0.63 |
| Residence | 1.52 | 0.66 |
| Frequency of listening to radio | 1.19 | 0.84 |
| Frequency of watching television | 1.60 | 0.62 |
| Employment status | 1.05 | 0.95 |
| Ever terminated a pregnancy | 1.01 | 0.98 |
| Birth interval (months) | 1.01 | 0.99 |
| Country | 1.07 | 0.93 |

**Table A2.** Demographic characteristics of respondents who switched from facility to home

|  | N | % |
| --- | --- | --- |
| **Age** |  |  |
| 15-19 | 217 | 5.71 |
| 20-24 | 1,159 | 30.52 |
| 25-29 | 1,068 | 28.12 |
| 30-34 | 695 | 18.3 |
| 35-39 | 455 | 11.98 |
| 40-44 | 168 | 4.42 |
| 45-49 | 36 | 0.95 |
| **Education level** |  |  |
| No education | 1,445 | 38.05 |
| Primary | 1,447 | 38.1 |
| Secondary | 859 | 22.62 |
| Higher | 47 | 1.24 |
| **Marital status** | |  |
| Married | 161 | 4.24 |
| Never in union | 2,668 | 70.25 |
| Living with a partner | 701 | 18.46 |
| Widowed | 44 | 1.16 |
| Divorced | 63 | 1.66 |
| Separated | 161 | 4.24 |
| **Wealth** |  |  |
| Poorest | 1,141 | 30.04 |
| Poorer | 905 | 23.83 |
| Middle | 891 | 23.46 |
| Richer | 584 | 15.38 |
| Richest | 277 | 7.29 |
| **Residence** |  |  |
| Urban | 1,004 | 26.43 |
| Rural | 2,794 | 73.57 |
| **Frequency of listening to radio** |  |  |
| Not at all | 1,879 | 49.47 |
| Less than once a week | 780 | 20.54 |
| At least once a week | 1,045 | 27.51 |
| Almost every day | 94 | 2.47 |
| **Frequency of watching television** |  |  |
| Not at all | 2,583 | 68.01 |
| Less than once a week | 465 | 12.24 |
| At least once a week | 643 | 16.93 |
| Almost every day | 107 | 2.82 |
| **Employment status** |  |  |
| Unemployed | 1,457 | 38.36 |
| Employed | 2,341 | 61.64 |
| **Ever terminated a pregnancy** |  |  |
| No | 3,286 | 86.52 |
| Yes | 512 | 13.48 |
| **Birth interval (months)** |  |  |
| <24 | 1,060 | 27.91 |
| 24 to 36 | 1,971 | 51.9 |
| >36 | 767 | 20.19 |

**Table A3.** Crude Odds Ratio (COR) of the factors associated with changes in location of childbirth among Sub-Saharan African women of reproductive age

|  | **Changed birth location** | **Changed from home to facility** |
| --- | --- | --- |
| **Variables** | **Model 1 - COR (95%CI)** | **Model 2 - COR (95%CI)** |
| **Age** |  |  |
| 15-19 | Ref | Ref |
| 20-24 | 0.98 (0.85 - 1.12) | 1.45 (1.16 - 1.82)** |
| 25-29 | 0.85 (0.74 - 0.98)* | 1.43 (1.15 - 1.79)** |
| 30-34 | 0.79 (0.69 - 0.92)** | 1.41 (1.13 - 1.75)** |
| 35-39 | 0.80 (0.69 - 0.93)** | 1.51 (1.20 - 1.89)*** |
| 40-44 | 0.80 (0.67 - 0.95)* | 1.26 (0.97 - 1.64) |
| 45-49 | 0.86 (0.65 - 1.13) | 1.20 (0.83 - 1.72) |
| **Education level** |  |  |
| No education | Ref | Ref |
| Primary | 1.14 (1.06 - 1.22)*** | 1.85 (1.67 - 2.05)*** |
| Secondary | 0.93 (0.85 - 1.01) | 2.89 (2.53 - 3.30)*** |
| Higher | 0.34 (0.26 - 0.44)*** | 4.77 (2.82 - 8.08)*** |
| **Marital status** |  |  |
| Married | Ref | Ref |
| Never in union | 0.88 (0.75 - 1.03) | 0.65 (0.51 - 0.83)*** |
| Living with partner | 0.93 (0.79 - 1.10) | 0.81 (0.63 - 1.05) |
| Widowed | 1.00 (0.74 - 1.37) | 0.70 (0.46 - 1.08) |
| Divorced | 0.81 (0.61 - 1.07) | 0.51 (0.33 - 0.81)** |
| Separated | 1.07 (0.86 - 1.33) | 0.83 (0.59 - 1.16) |
| **Wealth** |  |  |
| Poorest | Ref | Ref |
| Poorer | 0.99 (0.92 - 1.07) | 1.25 (1.12 - 1.38)*** |
| Middle | 1.02 (0.95 - 1.11) | 1.58 (1.40 - 1.79)*** |
| Richer | 0.85 (0.77 - 0.94)** | 1.96 (1.69 - 2.27)*** |
| Richest | 0.51 (0.45 - 0.57)*** | 3.11 (2.59 - 3.74)*** |
| **Residence** |  |  |
| Urban | Ref | Ref |
| Rural | 1.27 (1.17 - 1.39)*** | 0.50 (0.44 - 0.56)*** |
| **Frequency of listening to radio** |  |  |
| Not at all | Ref | Ref |
| Less than once a week | 1.10 (1.02 - 1.18)** | 1.73 (1.55 - 1.93)*** |
| At least once a week | 1.02 (0.96 - 1.10) | 2.13 (1.92 - 2.36)*** |
| Almost every day | 1.21 (0.93 - 1.59) | 1.76 (1.24 - 2.50)** |
| **Frequency of watching television** |  |  |
| Not at all | Ref | Ref |
| Less than once a week | 1.05 (0.96 - 1.14) | 2.06 (1.81 - 2.35)*** |
| At least once a week | 0.72 (0.66 - 0.78)*** | 2.27 (1.97 - 2.61)*** |
| Almost every day | 1.03 (0.80 - 1.32) | 2.37 (1.71 - 3.29)*** |
| **Currently working** |  |  |
| No | Ref | Ref |
| Yes | 1.02 (0.96 - 1.09) | 1.19 (1.09 - 1.30)*** |
| **Ever terminated a pregnancy** |  |  |
| No | Ref | Ref |
| Yes | 1.08 (1.00 - 1.17) | 1.28 (1.14 - 1.45)*** |
| **Birth interval (months)** |  |  |
| <24 | Ref | Ref |
| 24 to 36 | 1.25 (1.17 - 1.34)*** | 1.31 (1.19 - 1.44)*** |
| >36 | 1.23 (1.13 - 1.33)*** | 1.54 (1.37 - 1.72)*** |
| Observations | 61,240 | 24,538 |

**Note:** COR = Crude Odds Ratio. 95% confidence interval (CI) in parentheses Ref = Reference group. Sampling weights and clustering were accounted for in all estimations. *** p<0.001, ** p<0.01, * p<0.05

**
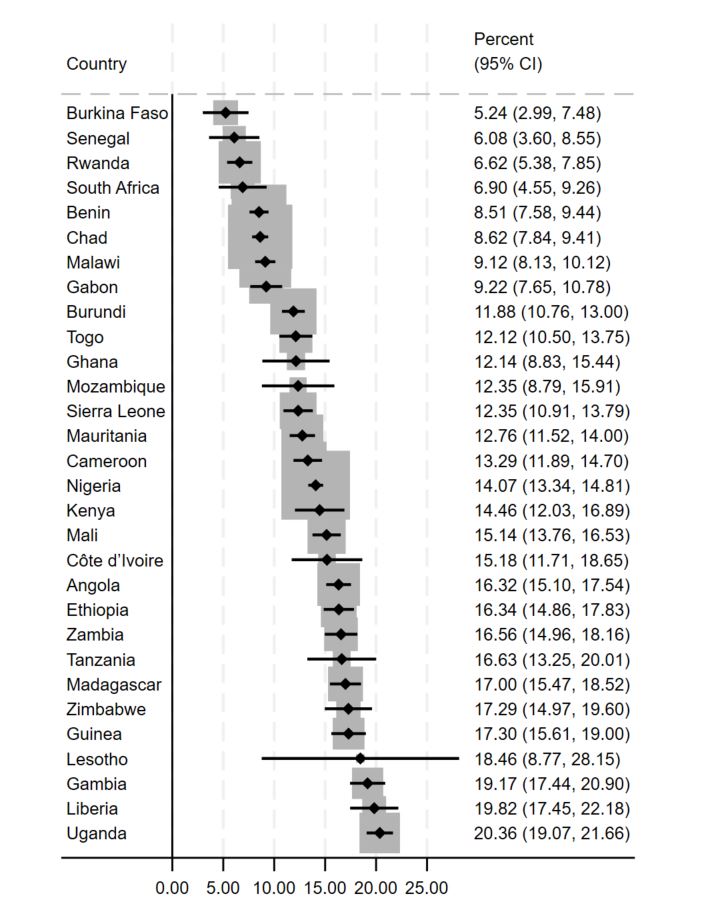
**

**Figure A1.** Forest plot of the percentage of women who changed childbirth location by country

**
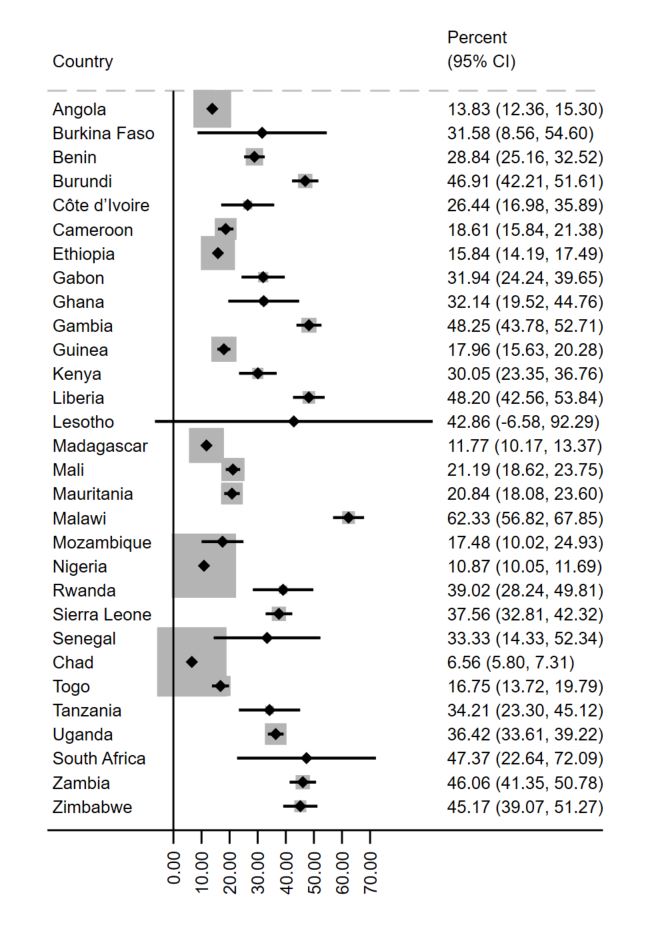
**

**Figure A2.** Forest plot of the percentage of women who shifted from home to facility for childbirth by country
